# Legal Notes

**Published:** 1899-12-16

**Authors:** 


### LEGAL NOTES.

### THE WORKMEN'S COMPENSATION ACT.

Two very interesting cases recently came before the Court of Appeal, in both of which a somewhat similar question arose, namely, how far a workman was entitled to compensation under the Workmen's Compensation Act, 1897, for injury received to a part of the body which was not itself free from disease or a tendency thereto; and, as the law was laid down with great clearness, it may be well to refer to it somewhat fully. In the first case, a workman who in the ordinary course of his work had to make a considerable exertion in doing a certain thing, namely, in starting a gas engine, suddenly on so doing vomited blood, which vomiting of blood caused his death. On post-mortem examination it was found that "there was evidence of congestion and inflammation." We quote the *Times* report: "Two of the doctors gave it as their opinion that the rupture of the blood vessel was caused by the strain, whereas another doctor stated his opinion that the strain apart from the disease did not cause the blood vessels to give way, and that, in his opinion, hæmorrhage from the stomach could not result from strain without disease." The County Court Judge decided in favour of the respondent, saying that "to make it an accident there must be something happening beyond the mere fact of the disease suddenly taking a fatal turn, *e.g.*, some break in the machinery, some fall of the workman, or something else out of the ordinary and normal course of the workman's employment." The appeal was dismissed.

In the second case the workman, who was a smith, had in the course of his work to hold a tool upon an anvil while another workman struck the flat part of it with a hammer. By accident, however, instead of striking the flat part he struck the round or rod part of the tool, and so jarred the workman's hand, and in consequence of this it became swollen and gout in the hand was brought on. Evidence was given by the doctor that he had previously attended the man for gout in the hand and elbow, that the workman had gouty diathesis, and that he thought the swollen hand was caused by gout brought on by the jar. The doctor's certificate stated that the man was suffering from a weak hand caused partially by the injury. The County Court Judge awarded the respondent 15s. a-week, stating that the respondent came within the Act. For the appellants it was contended that there was no evidence that the injury was caused solely by the accident. The gout, it was urged, was the cause of the swollen hand. In giving the decision of the Court, Lord Justice A. L. Smith said that the evidence showed that the mis-hit by the hammer jarred the respondent's hand. That mis-hit was an accident, and if it had broken the hand the case would have admittedly been within the Act. Any-one might have a weak hand. Was it to be said that, in such a case as this, only those with cast-iron hands came within the Act? The injury was caused by an accident, and the Act applied.

The teaching of these cases is very clear. Without an accident the Act will not apply. With an accident it will apply even though the morbid condition produced is largely the outcome of the diathesis of the sufferer. It is the accident which is the thing. If in the first case the man had tripped up, or the machinery had broken, or anything had happened of the nature of an accident in the performance of the work which he was doing, no doubt compensation would have been allowed, notwithstanding that his stomach was not healthy at the time. In the second case, if the "jar" had occurred as part of the man's ordinary work he could not have got compensation. Everything hinges on the condition being caused by an "accident."

